# The Association between the Use of Proton Pump Inhibitors and the Risk of Hypomagnesemia: A Systematic Review and Meta-Analysis

**DOI:** 10.1371/journal.pone.0112558

**Published:** 2014-11-13

**Authors:** Chan Hyuk Park, Eun Hye Kim, Yun Ho Roh, Ha Yan Kim, Sang Kil Lee

**Affiliations:** 1 Division of Gastroenterology, Department of Internal Medicine, Severance Hospital, Institute of Gastroenterology, Yonsei University College of Medicine, Seoul, Korea; 2 Biostatistics Collaboration Unit, Yonsei University College of Medicine, Seoul, Korea; University Hospital Llandough, United Kingdom

## Abstract

**Background:**

Although many case reports have described patients with proton pump inhibitor (PPI)-induced hypomagnesemia, the impact of PPI use on hypomagnesemia has not been fully clarified through comparative studies. We aimed to evaluate the association between the use of PPI and the risk of developing hypomagnesemia by conducting a systematic review with meta-analysis.

**Methods:**

We conducted a systematic search of MEDLINE, EMBASE, and the Cochrane Library using the primary keywords “proton pump,” “dexlansoprazole,” “esomeprazole,” “ilaprazole,” “lansoprazole,” “omeprazole,” “pantoprazole,” “rabeprazole,” “hypomagnesemia,” “hypomagnesaemia,” and “magnesium.” Studies were included if they evaluated the association between PPI use and hypomagnesemia and reported relative risks or odds ratios or provided data for their estimation. Pooled odds ratios with 95% confidence intervals were calculated using the random effects model. Statistical heterogeneity was assessed with Cochran’s Q test and *I* ^2^ statistics.

**Results:**

Nine studies including 115,455 patients were analyzed. The median Newcastle-Ottawa quality score for the included studies was seven (range, 6–9). Among patients taking PPIs, the median proportion of patients with hypomagnesemia was 27.1% (range, 11.3–55.2%) across all included studies. Among patients not taking PPIs, the median proportion of patients with hypomagnesemia was 18.4% (range, 4.3–52.7%). On meta-analysis, pooled odds ratio for PPI use was found to be 1.775 (95% confidence interval 1.077–2.924). Significant heterogeneity was identified using Cochran’s Q test (df = 7, *P*<0.001, *I* ^2^ = 98.0%).

**Conclusions:**

PPI use may increase the risk of hypomagnesemia. However, significant heterogeneity among the included studies prevented us from reaching a definitive conclusion.

## Introduction

Proton pump inhibitors (PPIs) are a mainstay of treatment for acid-related diseases, including gastroesophageal reflux disease, functional dyspepsia, and peptic ulcer disease [1]–[5]. PPIs have an excellent safety profile, and have become one of the most commonly prescribed class of drugs in both primary and specialty care [6]. However, PPI use may induce some adverse events, including interstitial nephritis [7], respiratory infections [8], *Clostridium difficile* colitis [9], and hip fractures [10]. More recently, it has been reported that hypomagnesemia may also be induced by PPIs. The association between symptomatic hypomagnesemia and PPI use was first described in two patients in 2006 [11]. Since this report, many case reports have accumulated, supporting the association between PPI use and induced hypomagnesemia [12]–[16]. The Food and Drug Administration issued a Drug Safety Communication in 2011 [17], emphasizing the importance of long term use of prescription PPIs in this association. Moreover, a systematic review of 18 case reports of PPI-induced hypomagnesemia found that discontinuation of PPI use resulted in recovery from PPI-induced hypomagnesemia [18]. Although the pathophysiology of PPI-induced hypomagnesemia has not been definitively determined, impaired absorption of intestinal magnesium, due to administration of PPIs, may be one possible mechanism [19]. Decreased luminal pH in the intestine, caused by PPI use, may alter the affinity of the transient receptor potential melastatin-6 and transient receptor potential melastatin-7 (TRPM6/7) channel for Mg^2+^, reducing active transport of Mg^2+^ [20], [21].

Although many case reports have described patients with PPI-induced hypomagnesemia, the impact of PPI use on hypomagnesemia has not been fully clarified in comparative studies [22]–[30]. In some studies, the association between PPI use and hypomagnesemia was not identified [23]–[25], [29], while in others, PPI use was found to increase the risk of hypomagnesemia [22], [26]–[28], [30]. The discrepancy between these studies may be attributed to various patient characteristics, and/or underlying conditions.

To examine this topic, we performed a systematic review with meta-analysis, of existing comparative studies that investigated the association between PPI use and the risk of developing hypomagnesemia.

## Materials and Methods

### Search strategy

We searched for all relevant studies published between January 1990 and May 2014 that examined the effect of PPI use on the risk of hypomagnesemia, using MEDLINE, EMBASE, and the Cochrane Library. The terms “proton pump,” “dexlansoprazole,” “esomeprazole,” “ilaprazole,” “lansoprazole,” “omeprazole,” “pantoprazole,” “rabeprazole,” “hypomagnesemia,” “hypomagnesaemia,” and “magnesium” were used in our search. We also examined the references of screened articles, in order to identify additional studies. All human studies published in English were considered, and the latest date for updating our search was August 19, 2014.

### Study selection

In the first stage of the study selection, the titles and abstracts of papers returned by our keyword search were examined to exclude irrelevant articles. Next, the full-text of all selected studies was screened, according to our inclusion and exclusion criteria. The inclusion criteria specified (1) studies regarding PPIs and hypomagnesemia, and (2) studies reporting relative risks or odds ratios (ORs) of PPI use, or provided data for their calculation. The exclusion criteria ruled out publications in a language other than English. Two investigators (C.H.P. and E.H.K.) independently evaluated studies for eligibility, resolved any disagreements through discussion and consensus. If no agreement could be reached, a third investigator (S.K.L.) decided eligibility.

To understand the risk of bias in individual studies, a formal quality assessment of studies was performed, along with subgroup analysis. The methodological quality of observational studies was independently assessed by two investigators (C.H.P. and E.H.K.), using the Newcastle-Ottawa scale [31], [32]. Using this scale, observational studies were scored across three categories: selection (4 questions), comparability of study groups (2 questions), and ascertainment of the exposure or outcome (3 questions). All questions were assigned a score of one point, with the exception of comparability of study groups, in which a maximum of two points were awarded. Studies with a cumulative score ≥7 were considered high quality [33], [34].

### Data extraction

Using a data extraction form developed in advance, two reviewers (C.H.P. and E.H.K.) independently extracted the following information: first author, year of publication, study design, country, enrollment period, study population, age and sex of patients, definition of hypomagnesemia, total number of patients in each group (exposed vs. not exposed), ORs, and 95% confidence intervals (CIs). When incomplete information was available, attempts were made to contact the corresponding authors of the studies for additional information.

### Outcomes assessed

Our primary analysis focused on assessing the risk of hypomagnesemia among users of PPIs. Our a priori hypothesis included study population (inpatients vs. others) as a potential explanation for heterogeneity in the direction and magnitude of effect. For exploratory analysis we conducted subgroup analysis according to the cut-off value of serum magnesium level (1.7 mg/dL vs. 1.8 mg/dL).

### Statistical analysis

Meta-analyses were performed to calculate pooled ORs with 95% CIs [35]. Taking a conservative approach, we used a random effects model, which produces wider CIs than a fixed effect model. We assessed heterogeneity using two methods: Cochran’s Q test, which was considered statistically significant for heterogeneity if *P*<0.1, and *I* ^2^ statistics, with values >50% suggestive of significant heterogeneity [36]. The tests for funnel plot asymmetry was not conducted when the included studies were less than 10 [37]. All *P*-values were 2-tailed, and a value of *P*<0.05 was considered statistically significant for all tests (except heterogeneity). Analysis and reporting were performed according to the Preferred Reporting Items for Systematic Reviews and Meta-Analyses (PRISMA) guidelines [38]. Statistical analyses were conducted using the statistical software Comprehensive Meta Analysis (version 2.2.064; Biostat Inc., Englewood, NJ, USA).

## Results

### Study selection

**Figure 1:**
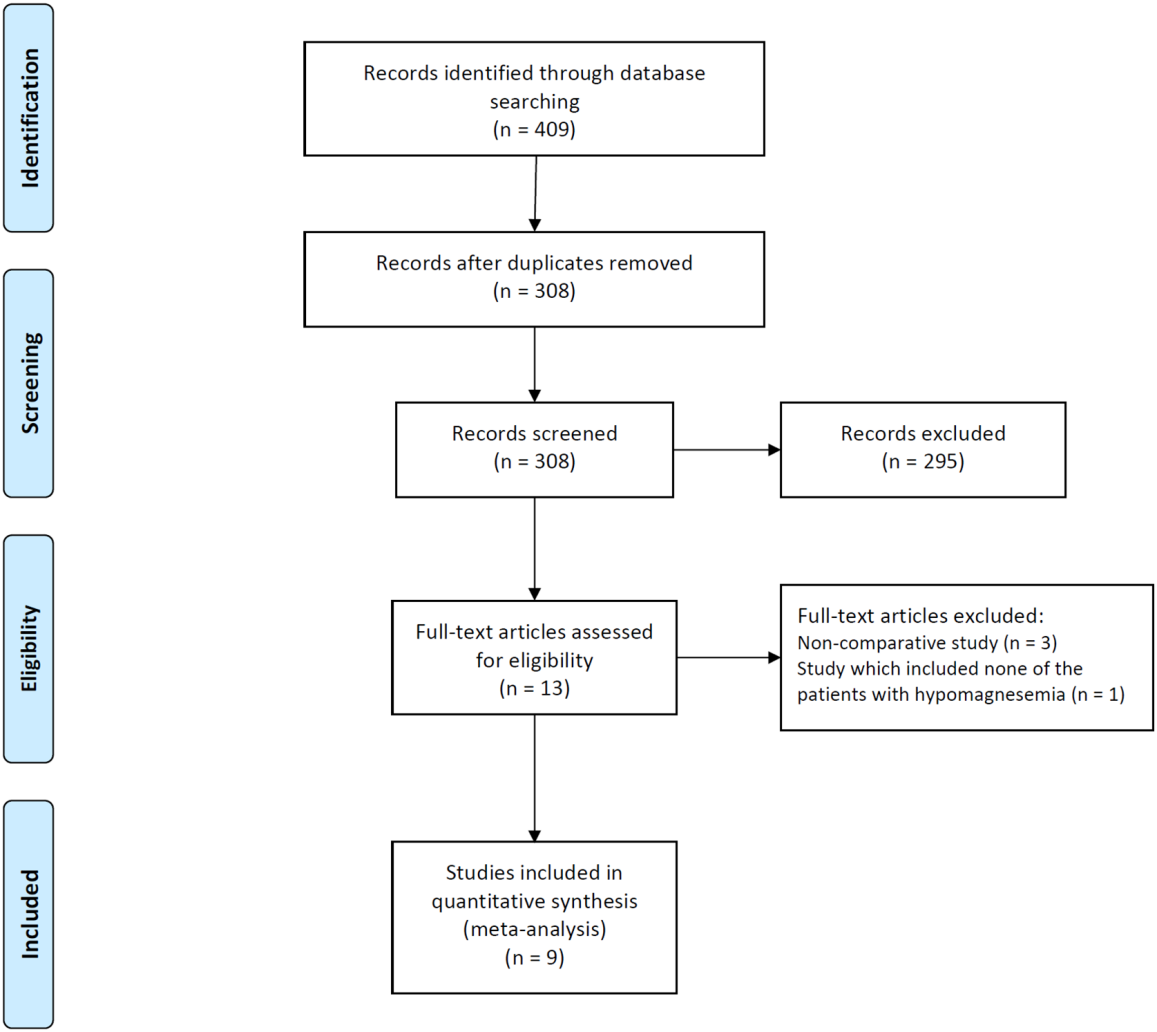
Flow diagram of studies included in the meta-analysis. PPI, proton pump inhibitor.

A flow diagram for our systematic review is shown in Fig. 1. In summary, 409 studies were identified by our literature search. After scanning titles and abstracts, we discarded 101 duplicate articles retrieved through multiple search engines. Another 295 irrelevant articles were excluded based on the titles and abstracts. The full texts of the 13 remaining articles were reviewed, and 4 non-pertinent articles were excluded. Of 4 excluded studies, three were non-comparative studies without control group and remaining one included none of the patients with hypomagnesemia in both treatment and control group.

Of the original 409 studies, nine studies were deemed appropriate for our meta-analysis [22]–[25], [27], [28]. These studies included a total of 115,455 patients, and all had been published in the past 2 years, between 2012 and 2014. The enrollment period for these studies ranged from 2000 to 2013, and all were retrospective in nature. Of the included studies, five included only hospitalized patients [22]–[25], [30], two included outpatients only [28], [29], one included both inpatients and outpatients [26], and one included patients with end-stage renal disease on hemodialysis, but did not provide data regarding the hospitalization of patients [27]. The median Newcastle-Ottawa quality score for these studies was seven (range, 6–9), and all studies except one were considered high quality. The characteristics of the studies included in the meta-analysis are summarized in Table 1.

**Table 1:** Characteristics of studies included in the meta-analysis.

Author	Year of	Study design	Country	Enrollment	Study population	Number of		Study quality	
	publication			period	(Inpatients vs. Out patients)	patients	Selection	Comparability	Exposure/Outcome
Gau *et al.* [22]	2012	Cross-sectional	USA	2004–2008	Inpatients	487	***	**	**
El-Charabaty *et al.* [23]	2013	Cross-sectional	USA	2007–2012	Inpatients^a^	421	***	*	**
Koulouridis *et al.* [24]	2013	Case-control	USA	2000–2007	Inpatients	804	***	**	***
Danziger *et al.* [25]	2013	Cross-sectional	USA	2001–2008	Inpatients^b^	11,490	***	**	**
Kim *et al.* [26]	2013 (in press)	Retrospective cohort	Korea	2007–2012	Inpatients & Outpatients	1,356	***	**	***
Alhosaini *et al.* [27]	2014	Retrospective cohort	USA	2012–2013	NA^c^	62	***	**	***
Markovitis *et al.* [28]	2014 (in press)	Cross-sectional	Israel	2008–2011	Outpatients	95,205	****	**	***
Van Ende *et al.* [29]	2014 (in press)	Cross-sectional	Belgium	2004–2006	Outpatients^d^	512	***	**	**
Lindner *et al.* [30]	2014 (in press)	Cross-sectional	Switzerland	2009–2010	Inpatients	5,118	***	**	**

Asterisk represents quality scores based on the Newcastle-Ottawa scale.

a Patients who admitted for unstable angina, non-ST elevation myocardial infarction, and ST elevation myocardial infarction were included only.

b Patients who admitted to intensive care unit were included only.

c Patients with end-stage renal disease on hemodialysis were included only.

d Renal transplant recipients were included only.

### Risk of hypomagnesemia

**Figure 2:**
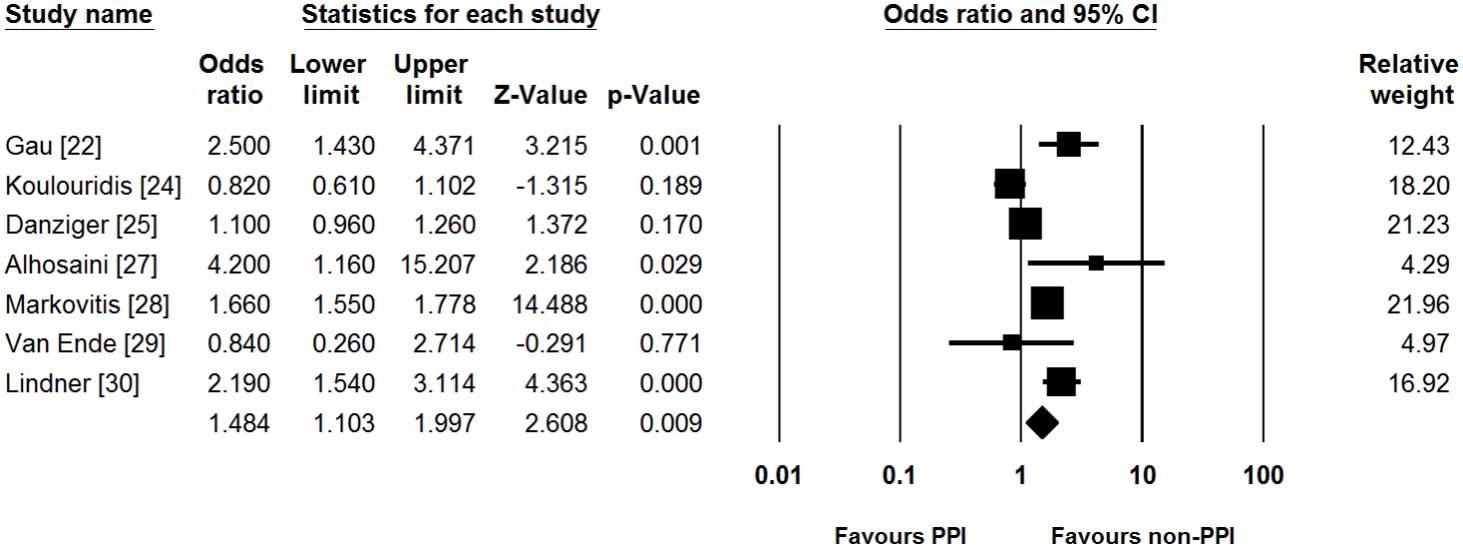
Forest plots for risk of hypomagnesemia. PPI, proton pump inhibitor; CI, confidence interval.

The cut-off value of serum magnesium level for defining hypomagnesemia was 1.6, 1.7, and 1.8 mg/dL in one [25], five [22], [24], [26], [28], [29], and three studies [23], [27], [30], respectively (Table 2). Among patients taking PPIs, the median proportion of patients with hypomagnesemia in all included studies was 27.1% (range, 11.3–55.2%). In addition, the median of the proportion of patients with hypomagnesemia in those not taking PPIs was 18.4% (range, 4.3–52.7%) across the studies. Of 9 included studies, six reported both unadjusted OR and adjusted OR. Other two studies showed unadjusted OR only. Remaining one study showed adjusted OR only. Most studies adjusted for the following confounders: use of diuretics (6/7), renal function (5/7), age (4/7), diabetes mellitus (4/7), and comorbidities (4/7). On meta-analysis, pooled unadjusted OR for PPI use was found to be 1.775 (95% CI = 1.077–2.924). Significant heterogeneity was identified (Cochran’s Q test, df = 7, *P*<0.001, *I* ^2^ = 98.0%). This risk increase with PPI use persisted even after adjusting for potential confounders where reported in studies (pooled adjusted OR [95% CI] = 1.484 [1.103–1.997], Fig. 2), although the heterogeneity persisted (Cochran’s Q test, df = 6, *P*<0.001, *I* ^2^ = 89.1%).

**Table 2:** Characteristics of individuals in included studies.

Author	Age, mean ± SD			Male, n (%)		Definitionof	HypoMg inPPI users		HypoMg innon-PPI users		UnadjustedOR	AdjustedOR	Adjusted variables
	HypoMg	Non-HypoMg		HypoMg	Non-HypoMg	hypoMg	HypoMg	PPI user	HypoMg	PPI user	(95% CI)	(95% CI)	
						mg/dL	n (%)	N	n (%)	n			
Gau *et al.* [22]	73.3±11.5	76.3±12.1		23(29.5)	150 (36.7)	<1.7	48 (23.2)	207	30(10.7)	280	2.52(1.53–4.14)	2.50(1.43–4.36)	Age, sex, DM, past medication history of congestive heart failure, diuretics, supplementation of potassium and magnesium, discharge diagnosis of any acute gastrointestinal illness, serum albumin, serum potassium, and serum creatinine.
El-Charabaty*et al.* [23]	NA	NA		NA	NA	<1.8	47 (25.5)	184	48(20.3)	237	1.35(0.85–2.14)	NA	
Koulouridis*et al.* [24]	70.0±14.4	70.0±14.4		161(40.0)	161 (40.0)	<1.7	219 (47.9)	457	183(52.7)	347	0.82(0.62–1.09)	0.82(0.61–1.11)	Charlson-Deyo comorbidity index, DM, GERD, diuretics, and eGFR.
Danziger*et al.* [25]	PPI group: 67.8±15.4 H_2_ blocker group: 66.9±15.9 Control group: 61.1±19.2			PPI group:1,403 (53.3) H_2_ blocker group: 368 (56.3) Control group: 4,796 (58.5)		<1.6	405 (15.4)	2,632	1,456(16.4)	8,858	0.92(0.82–1.04)	1.10(0.96–1.25)	Age, sex, ethnicity, comorbidities, diuretics, renal function, systolic blood pressure, heart rate, temperature, serum calcium, serum phosphate, serum glucose, and serum hematocrit.
Kim *et al.* [26]	PPI group: 56.2±14.3 Control group: 55.2±14.1			PPI group: 65(58.0) Control group:462 (37.1)		≤1.7	32 (28.6)	112	177(14.2)^a^	1,244	2.41(1.55–3.74)	NA	
Alhosaini*et al.* [27]	64.8±7.2		64.0±9.6	24(100.0)	38(100.0)	<1.8	16 (55.2)	29	8(24.2)	33	3.85(1.30–11.34)	4.20(1.16–15.2)	Age, DM, diuretics, plasma albumin, protein intake, duration of dialysis, and dialytic urea clearance.
Markovitis*et al.* [28]	58.1±18.5		47.4±20.3	2,031(35.7)	33,003(36.9)	≤1.7	2,532 (11.3)	22,458	2,890(4.1)	69,714	2.94(2.78–3.11)	1.66(1.55–1.78)	Age, sex, comorbidities(hypertension, DM, heart failure, and malignancy), eGFR, drugs (diuretics, imunosuppressants, lithium, and digoxin), and recent hospitalization.
Van Ende *et al.* [29]	NA		NA	NA	NA	<1.7	NA	101	NA	411	NA	0.84(0.26–2.71)	Hemoglobin, tacrolimus, vitamin D substitution, and eGFR.
Lindner *et* *al.* [30]	56±20		54±20	798 (66)	2,565 (64)	<1.8	155 (36.6)	423	1,091 (23.2)	4,695	1.91(1.55–2.35)	2.19(1.54–2.86)	Charlson comorbidity index score, diuretics, and eGFR.

a This value was obtained through contact with the corresponding author of the study.

HypoMg, hypomagnesemia; PPI, proton pump inhibitor; DM, diabetes mellitus; GERD, gastroesophageal reflux disease; eGFR, estimated glomerular filtration rate; SD, standard deviation; OR, odds ratio; CI, confidence interval; NA, not available.

### Subgroup analysis

**Figure 3:**
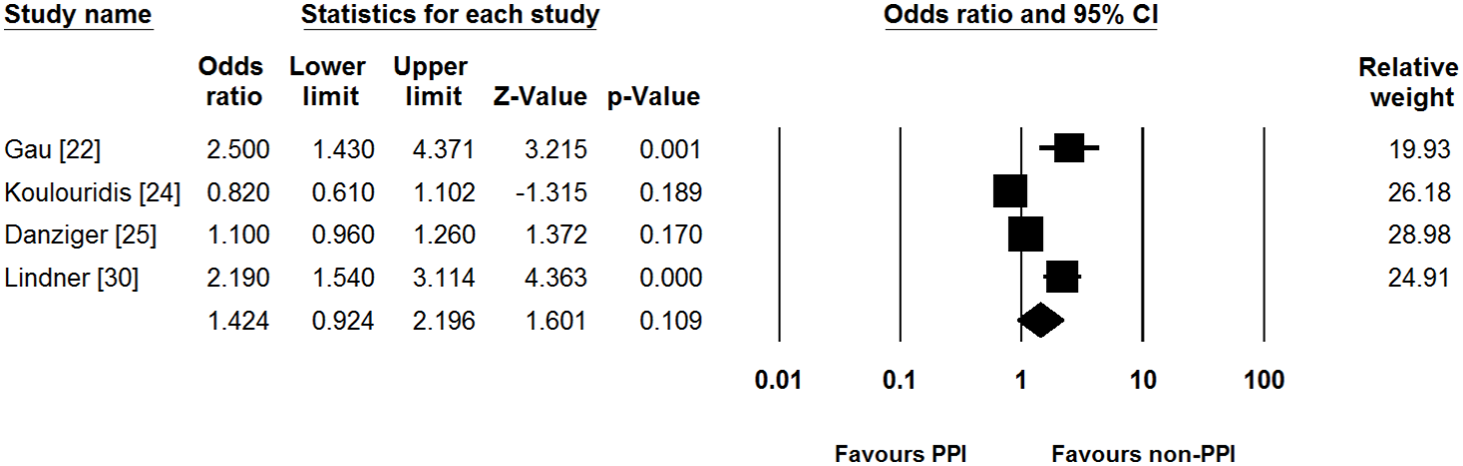
Subgroup analysis for studies which included only hospitalized patients. PPI, proton pump inhibitor; CI, confidence interval.

We performed pre-planned subgroup analysis of studies, based on the hospitalization of patients. In five studies which included only hospitalized patients [22]–[25], [30], PPI use was not associated with hypomagnesemia (pooled unadjusted OR [95% CI] = 1.342 [0.895–2.011]). Significant heterogeneity did exist in the inpatient subgroup (Cochran’s Q test, df = 4, *P*<0.001, *I* ^2^ = 92.0%). Pooled adjusted OR showed similar results between PPI use and incidence of hypomagnesemia compared to pooled unadjusted OR (pooled adjusted OR [95% CI] = 1.424 [0.924–2.196], Fig. 3). Significant heterogeneity persisted in this analysis (Cochran’s Q test, df = 3, *P*<0.001, *I* ^2^ = 88.3%).

**Figure 4:**
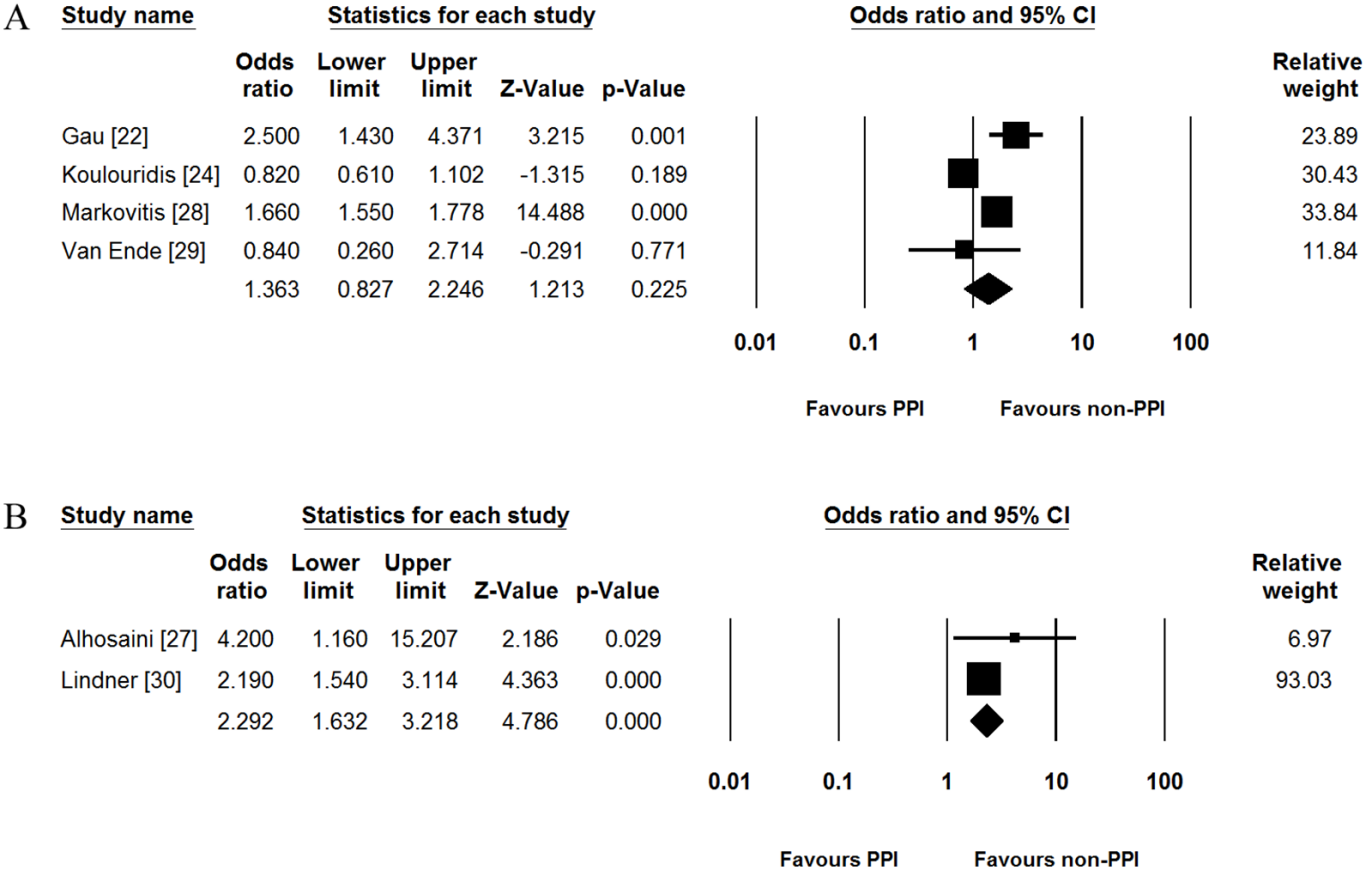
Subgroup analysis according to the cut-off value of serum magnesium levels. (A) Subgroup analysis for studies based on the 1.7 mg/dL of cut-off value. (B) Subgroup analysis for studies based on the 1.8 mg/dL of cut-off value. PPI, proton pump inhibitor; CI, confidence interval.

For exploratory analysis, we performed additional subgroup analysis according to the cut-off value of serum magnesium level. In 4 studies which reported adjusted OR based on the 1.7 mg/dL of cut-off value [22], [24], [28], [29], PPI use was not associated with incidence of hypomagnesemia (pooled adjusted OR [95% CI] = 1.363 [0.827–2.246], Fig. 4A). Significant heterogeneity was identified in this subgroup (Cochran’s Q test, df = 3, *P*<0.001, *I* ^2^ = 87.7%). In 2 studies which showed adjusted ORs based on the 1.8 mg/dL of cut-off value [27], [30], on the contrary, PPI use increased the risk of hypomagnesemia (pooled adjusted OR [95% CI] = 2.292 [1.632–3.218], Fig. 4B). There was no heterogeneity in this subgroup (Cochran’s Q test, df = 1, *P* = 0.339, *I* ^2^ = 0.0%).

## Discussion

Intracellular magnesium is an important cofactor for enzymatic reactions, and is critical for energy metabolism involving adenosine triphosphate [19]. Magnesium homeostasis is determined primarily by two processes, gastrointestinal absorption and renal excretion [39]. Gastrointestinal magnesium absorption occurs through both passive paracellular movement, and active transport into the portal venous system [19]. Active magnesium transport in the gut occurs through the combined action of TRPM6/7 channels, which are present in the apical membrane of enterocytes [39]. Previous studies have found that renal excretion of magnesium is reduced appropriately in patients with PPI-induced hypomagnesemia [40], [41], and that a PPI-induced decrease in the luminal pH of the intestine might alter the TRPM6/7 channel’s affinity for magnesium [21]. Therefore, impaired intestinal absorption rather than renal excretion is considered the primary cause of PPI-induced hypomagnesemia.

Many previous case reports [12]–[16], and proposed mechanisms of PPI-induced hypomagnesemia, have promoted awareness of the risk of hypomagnesemia in patients taking PPIs. However, the risk for PPI-induced hypomagnesemia should be evaluated by comparative studies, since the prevalence of hypomagnesemia is not rare. One previous population-based study found that 2% of subjects (104 of 5,179) have hypomagnesemia [42]. Other population-based studies also have suggested that hypomagnesemia is not rare in the general population [43], [44]. Moreover, in hospitalized patients, electrolyte disorders (including hypomagnesemia) are often acute and severe. Therefore, case reports alone cannot analyze the effect of PPI use on hypomagnesemia, and well-designed, comparative studies are needed to clarify the risk of PPI-induced hypomagnesemia.

Our meta-analysis showed statistical significance between PPI use and the risk of hypomagnesemia. The risk of hypomagnesemia in PPI users persisted even after adjusting for confounding variables. However, the significant heterogeneity among the included studies may be a concern. The significance of heterogeneity implied that the effect of PPI use on hypomagnesemia was varied. The observed heterogeneity may be due to the various types of study design and population among our included studies. For example, Gau *et al.*’s study [22], Koulouridis *et al.*’s study [24], and Lindner *et al.*’s study [30] included hospitalized patients, regardless of disease type. However, Koulouridis *et al.*’s study was designed as case-control study, while other two studies were designed as cross-sectional study. In contrast, Danziger *et al.*’s study [25] included only patients admitted to intensive care units. El-Charabaty *et al.*’s study [23] included only hospitalized patients with unstable angina, non-ST elevation myocardial infarction, and ST elevation myocardial infarction. Kim *et al.*’s study [26] included both inpatients and outpatients. Alhosaini *et al.*’s study [27] included only patients with end-stage renal disease on hemodialysis. Van Ende *et al.*’s study [29] included only renal transplant recipients. Finally, Markovitis *et al.*’s study [28] included only outpatients in the community setting. In addition, the definition of hypomagnesemia was varied among the included studies. These variations in study design may be reflected in the proportion of patients with hypomagnesemia for each group (patients taking PPIs vs. those not taking PPIs). The proportion of patients with hypomagnesemia ranged from 11.3% to 55.2% (PPI user group), and from 4.1% to 52.7% (non-PPI user group), and therefore, we believe that the incidence or prevalence of hypomagnesemia may depend on the study design.

One of the clinical concerns on PPI-induced hypomagnesemia is whether the test for serum magnesium level should be performed before initiating PPIs or not. Although it has not been fully evaluated, experts usually recommend the test for serum magnesium level prior to initiation of PPIs when patients are expected to be on treatment for long period of time [45], [46]. While short-term standard dose PPI treatment has low risk, long-term PPI use may complicate health conditions in high risk patients for hypomagnesemia including elderly patients [46]. The inter-study differences in the proportion of patients with hypomagnesemia in our meta-analysis may suggest that there are patients with increased susceptibility to PPI-induced hypomagnesemia. Considering the excellent safety profile of PPIs, risk identification and stratification for PPI-induced hypomagnesemia may be more helpful for clinical practice, rather than investigation of PPI-induced hypomagnesemia in the general population. Kim *et al.*’s study, which evaluated associated factors for hypomagnesemia, found that long-term PPI use (>1 year), young age (<45 years), and concurrent cisplatin or carboplatin use were associated with hypomagnesemia in PPI users [26]. However, the study only included PPI users whose serum magnesium levels were available, and therefore, selection bias may be a concern. A population based study with regular checkup for serum magnesium level may be needed for clarifying the high risk patients for PPI-induced hypomagnesemia.

In our study, we conducted the two types of subgroup analysis. The former was subgroup analysis for studies which included hospitalized patients only, while the latter was subgroup analysis according to the cut-off value of serum magnesium level. In the former subgroup analysis, statistical significance between PPI use and the risk of hypomagnesemia was not shown. Although the subgroup included only hospitalized patients, the variation among the studies was still existed. In the latter subgroup analysis, PPI use increased the risk of hypomagnesemia in the studies whose cut-off value was 1.8 mg/dL rather than 1.7 mg/dL. These results implied that PPI-induced hypomagnesemia might not be as severe as we were concerned; however, further studies would be needed for assessing the severity of PPI-induced hypomagnesemia.

Although this is the first meta-analysis which demonstrated that PPI use could increase the risk of hypomagnesemia, there are several limitations. First, this is the meta-analysis for observational studies rather than randomized controlled trials. Magnesium assessment in a large database is usually healthcare-driven and potentially biased. In addition, dyspepsia may lead to prescribing PPIs as well as deficient food intake with low magnesium ingestion. Furthermore, serum magnesium was not usually evaluated in clinical practice. Although we conducted a meta-analysis using the adjusted ORs in the included studies, potential issues of confounding variables may be exist. The conclusion from the meta-analysis for observational studies should be interpreted carefully. Second, the significant heterogeneity among the included studies was additional obvious limitation. Through our systematic review and meta-analysis, we found that the proportion of patients with hypomagnesemia depended on the study settings including patient population and characteristics. Prospective cohort studies will be needed to evaluate severity of PPI-induced hypomagnesemia and to identify high risk group for PPI-induced hypomagnesemia. Third, we could not assess the risk of PPI-induced hypomagnesemia according to the amount or duration of usage of PPIs because available data were limited in the included studies.

Despite of these limitations, our meta-analysis showed that PPI use may increase the risk of hypomagnesemia. However, significant heterogeneity among the included studies prevented us from reaching a definitive conclusion. Well-designed, prospective cohort studies, which include regular serum magnesium monitoring, would provide more conclusive results.

## Supporting Information

Checklist S1 **PRISMA checklist of the study.**
(DOC)
